# Measurement of Energy Intake Using the Principle of Energy Balance Overcomes a Critical Limitation in the Assessment of Energy Availability

**DOI:** 10.1186/s40798-023-00558-8

**Published:** 2023-02-22

**Authors:** Caroline A. Tarnowski, Sophie L. Wardle, Thomas J. O’Leary, Robert M. Gifford, Julie P. Greeves, Gareth A. Wallis

**Affiliations:** 1 School of Sport, Exercise and Rehabilitation Sciences, College of Life and Environmental Sciences, University of Birmingham, Birmingham, B15 2TT UK; 2 Army Health and Performance Research, Army Headquarters, Andover, UK; 3 Division of Surgery and Interventional Science, Department of Targeted Intervention, University College London, London, UK; 4 British Heart Foundation Centre for Cardiovascular Science, Queen’s Medical Research Institute, University of Edinburgh, Edinburgh, UK; 5 Research and Clinical Innovation, Royal Centre of Defence Medicine, Birmingham, UK; 6 Norwich Medical School, University of East Anglia, Norwich, UK

**Keywords:** Low energy availability, Relative energy deficiency in sport, Female and male athlete triad, Exercise, Nutrition, Athlete

## Abstract

Prolonged low energy availability, which is the underpinning aetiology of the Relative Energy Deficiency in Sport and the Female and Male Athlete Triad frameworks, can have unfavourable impacts on both health and performance in athletes. Energy availability is calculated as energy intake minus exercise energy expenditure, expressed relative to fat free mass. The current measurement of energy intake is recognized as a major limitation for assessing energy availability due to its reliance on self-report methods, in addition to its short-term nature. This article introduces the application of the energy balance method for the measurement of energy intake, within the context of energy availability. The energy balance method requires quantification of the change in body energy stores over time, with concurrent measurement of total energy expenditure. This provides an objective calculation of energy intake, which can then be used for the assessment of energy availability. This approach, the Energy Availability - Energy Balance (EA_EB_) method, increases the reliance on objective measurements, provides an indication of energy availability status over longer periods and removes athlete burden to self-report energy intake. Implementation of the EA_EB_ method could be used to objectively identify and detect low energy availability, with implications for the diagnosis and management of Relative Energy Deficiency in Sport and the Female and Male Athlete Triad.

## Key Points

- Prolonged low energy availability, which is the underpinning aetiology of the Relative Energy Deficiency in Sport and the Female and Male Athlete Triad frameworks, can have unfavourable impacts on both health and performance in athletes.
- The traditional measurement of energy intake, important for calculating energy availability, is recognized as a major limitation due to its reliance on self-report methods and short-term nature.
- An alternative approach is the Energy Availability - Energy Balance (EA_EB_) method which increases the reliance on objective measurements for assessing energy intake, provides an indication of energy availability status over longer periods, and removes athlete burden to self-report dietary intake.

## Introduction

Relative Energy Deficiency in Sport (RED-S) and the Female and Male Athlete Triad frameworks are highly topical within sports science and sports medicine due to the impact of low energy availability on athlete health and performance [1–4]. Low energy availability is defined as insufficient dietary energy available for maintenance of normal physiological functioning after the energy costs of exercise have been met [5]. Energy availability (EA) is thus calculated as Energy Intake (EI) minus Exercise Energy Expenditure (EEE) and is commonly expressed relative to Fat Free Mass (i.e., kcal⋅kg FFM⋅day^−1^). Research investigating changes in endocrine parameters over a short period of time suggests that an EA of ≥ 45 kcal⋅kg FFM⋅day^−1^ is considered ‘optimal’, with < 30 kcal⋅kg FFM⋅day^−1^ and between 30–45 kcal⋅kg FFM⋅day^−1^ classified as ‘clinically low EA’ and ‘subclinical/reduced EA’, respectively [6]. It should be acknowledged, however, that the existence of these defined thresholds is widely debated [7]. Nonetheless, EA is still a widely used concept, but there is no consensus on methods to measure each component and there are limitations in the methods typically used for its assessment [8]. These limitations could have substantial consequences for the interpretation of research on, and the use of EA as a practical tool to monitor, the health of athletes.

Measurement of EI is a recognized major limitation for assessing EA. Traditionally, EI is determined using prospective or retrospective self-report measures such as weighed food records and dietary recall. Such methods are prone to underreporting, changes in habitual dietary intake to ease the burden of self-reporting, and under-/over-reporting of certain foods [9, 10]. The athlete/participant burden of these methods also discourages assessments over longer timeframes, and studies are biased towards acute EA of 3–7 days even though the health consequences of low EA are likely to develop with chronic exposure.

The challenge of accurately assessing EI (and EEE, discussed elsewhere [11]), particularly in field settings, has resulted in the use of questionnaires (e.g., Low Energy Availability in Females Questionnaire [LEAF-Q]) and biomarkers proposed to reflect EA status (e.g., endocrine, physiological, metabolic) [1, 12, 13]. These methods are not without limitation. Questionnaires may be a useful screening, but not diagnostic, tool for low EA [13]. Biomarkers may be sensitive to factors other than EA, and some evidence suggests no biomarker relation to EA [14], making it difficult to draw firm conclusions, especially when only a single measurement is made [15]. To hold genuine practical utility, questionnaires and biomarkers need validation against an EA measurement that is not compromised by the self-report or short-term nature of its component parts.

This paper introduces the application of the Energy Balance (EB) method for measurement of EI within the context of EA. Measurement of the exercise component of EA has been considered recently elsewhere [11]. The EB method overcomes the subjective and short-term nature of current approaches to measuring EI, which enables a quantifiable, representative assessment of EA with favourable implications for both research and practice.

## The Principle of Energy Balance Objectively Measures Energy Intake

**Fig. 1:**
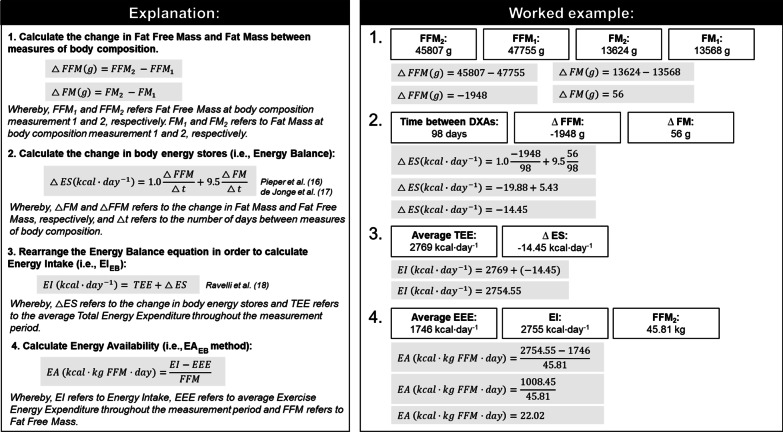
Calculating EA using the EA_EB_ method

Energy balance is equivalent to the change in body energy stores (∆ES) over time and can be assessed by multiplying changes in Fat Mass (FM) and Fat Free Mass (FFM) by their known metabolizable energy densities (i.e., 9.5 kcal⋅g^−1^ and 1.0 kcal⋅g^−1^, respectively) [16, 17]. Quantification of changes in body composition (∆ES) allows determination of net EB and with the additional measurement of Total Energy Expenditure (TEE), the EB method has been well established for the objective measurement of EI (TEE + ∆ES; described in Fig. 1) [18]. The precision of calculated EI is dependent on the methods used to measure ∆ES and TEE. A highly repeatable method for body composition analysis is required to accurately quantify ∆ES. Studies that have validated the EB method have mostly used Dual Energy X-ray Absorptiometry (DXA) for body composition, and Doubly Labelled Water (DLW) for free-living TEE [18, 19]. The accuracy of EI is poor when ∆ES is measured over a short-term period of days to weeks [17], but over a longer period (i.e., months), the ∆ES outweighs measurement error as the time between body composition measurements increases, and the accuracy of calculated EI using the EB method approaches 1% [17, 18, 20].

The use of the EB method to objectively measure EI has primarily been adopted by researchers in the obesity field [21–23]. A limited number of studies have used the method to determine EB in athletes throughout a competitive season [24–26], and one subsequently objectively calculated EI [27]. Where EI measurement in athletes is required, however, current consensus remains focused on improving accuracy and validity of traditional methods [28]. Thus, despite the potential to obtain objective measures of EI, the EB method is not widely recognised in sports medicine. By fully appreciating the utility and application of the EB method, we contend that significant improvements assessment of prolonged EA can be achieved.

## Application of the EB Method to Improve the Assessment of Energy Availability

The EB method quantifies EI (EI_EB_) and improves the calculation of EA by obviating the reliance on self-report approaches. This approach, the EA_EB_ method, is described in Fig. 1.

We tested the potential application of the EA_EB_ method using a published data set [11]. The participants were women completing 44 weeks of basic military training (N = 47). Throughout the study, there were three consecutive ‘terms’ that lasted ~ 14 weeks, each of which had a 10-day measurement period representative of the activities of the entire term. During the 10-day period, EEE from Moderate to Vigorous Physical Activity (MVPA) was measured using tri-axial accelerometery, and TEE was measured using DLW. In addition, EI was assessed traditionally using both 24-h food diaries and weighed food records. At the beginning (i.e., week 0) and end of each term (i.e., week 14), body composition was assessed using DXA. Using these variables, we calculated EA for one 14-week term traditionally (EA_TRA_) using traditionally measured EI (EI_TRA_) i.e., food diaries and weighed food records, and objectively (EA_EB_ method) using EI calculated via the EB method (EI_EB_). Due to some missing datapoints, EI values were compared for *N* = 38 and EA values were compared for *N* = 26. Conceptually, a previous study has adopted a similar approach [27]; however, EEE was estimated, which reduced the accuracy of assessment of EA. This *Current Opinion* provides the first published data set to use directly measured TEE, EB and EEE to calculate EA (i.e., the EA_EB_ method).

**Fig. 2:**
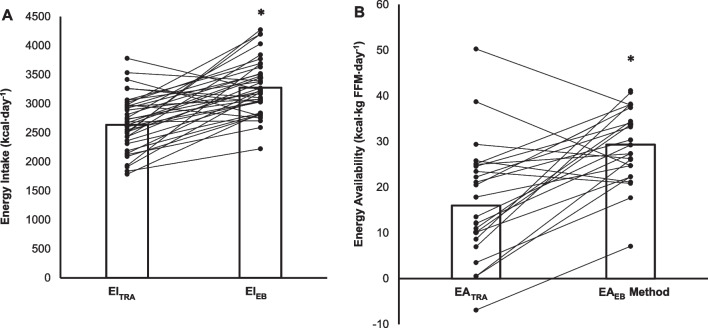
Energy Intake (**A**) and Energy Availability (**B**) calculated traditionally and objectively. Data are for N = 38 and N = 26, respectively, and are displayed as means and individual data points. *Denotes significantly different (P < 0.05). EI_TRA_ traditional energy intake method, EI_EB_ energy intake calculated using the energy balance method, EA_TRA_ traditional energy availability method, EA_EB_ energy availability - energy balance method

Previously, we strongly suspected that EI_TRA_, and subsequently EA_TRA_, were underestimated [11]. In the present analysis, EI_EB_ resulted in significantly higher values compared with EI_TRA_ (3276 ± 468 vs 2637 ± 481 kcal⋅day^−1^, *P* < 0.001; data are presented as mean ± SD and were compared by paired samples t-test, Fig. 2A). Consequently, significantly higher EA values were observed with the EA_EB_ method (29.3 ± 8.1 kcal⋅kg FFM⋅day^−1^) compared with EA_TRA_ (16.0 ± 12.5 kcal⋅kg FFM⋅day^−1^, *P* < 0.001, Fig. 2B). The higher EI_EB_ values are more plausible, which is supported by comparing EI_EB_ and EI_TRA_ values with TEE. For example, on average body mass did not significantly change during the term, which suggests participants were in energy balance (i.e., EI similar to TEE); but EI_TRA_ was on average 20 ± 16% (− 690 ± 554 kcal⋅day^−1^) lower than TEE. This percentage underestimation is in line with previous literature [10]. Conversely, EI_EB_ was on average only 3 ± 5% (− 81 ± 170 kcal⋅day^−1^) lower than TEE. In addition, as stated in our previous publication, the EA_TRA_ values were well below the purported low EA threshold of 30 kcal⋅kg FFM⋅day^−1^ [5] whereas the EA_EB_ method values were, on average, much closer to this threshold. The EA_EB_ method would mitigate against the risks of both under- and over-reporting of EI, of which the former is commonly assumed to affect the assessment of EA_TRA_, and both of which bring additional risk of failure to detect inadequate EA. We propose that the application of the EB method to determine EI improves the assessment of EA.

## Considerations for the EA_EB_ Method

### Advantages and Applications

The EA_EB_ method proposes an alternative approach to calculating EA with the advantage of using an objective measure of EI, which removes burden from the athlete to self-report EI, and minimises the resulting behaviour change from recording dietary intake [29]. In addition, the EA_EB_ method can measure EA status over a prolonged period (i.e., weeks to months) in a standardized manner. These advantages lend the EA_EB_ method to several applications. The EA_EB_ method provides an indication of prolonged EA status, which may be more relevant for detecting low EA than single short-term time-point assessments. This approach will improve intraindividual (e.g., across a season) and interindividual (e.g., different sports or playing positions) comparisons. Depending on the resources available, the approach could also be feasibly incorporated into routine monitoring practices of athletes, and provide complementary information for athlete support personnel in their endeavours to prevent the development of RED-S. The EA_EB_ method could provide a more accurate approach for prescribing recovery from RED-S by indicating how much EI needs to increase, or whether EEE needs to decrease [30]. The method would provide greater opportunity to robustly investigate the proposed EA thresholds. It is assumed that specific physiological disruptions occur below 30 kcal⋅kg FFM⋅day^−1^ but the threshold at which these disruptions occur varies widely between individuals [31]. Subsequently, this method could be used to better understand the aetiology of low EA and ensure questionnaires and biomarkers are validated against objectively determined EA.

### Practicalities and Limitations

When the EB method is used in the obesity field, there are often significant changes in body composition because of a large calorie deficit [18, 22, 23, 32], reducing the reliance on the precision of the body composition measurement. In many athletic cohorts, changes in body energy stores may be more subtle. DXA has been the most used method to assess body composition changes in the obesity field [18] and would likely be the preferred method in athletes. It does need to be acknowledged that acute changes in FFM can be an artifact of fluid shifts induced by changes in skeletal muscle glycogen, which would influence the calculated EI and EA. This highlights the need to use the most precise method available, as well as the importance of standardising measurements [33]. Methods such as Bioelectrical Impedance Analysis (BIA) or skinfold thickness may be more readily available in an athletic context but are not as accurate at measuring changes in FM or FFM [34–36].

The optimal time between body composition measures is inconclusive. Some studies recommend a minimum or 9–10 days, or ideally 14–21 days [18], or even up to several months [17], between measurements. Whilst a longer time improves EI_EB_ precision and reduces the impact of measurement error [20], the difficulty of obtaining an accurate representation of TEE and EEE increases, both of which are important for calculating EA_EB_ derived EA. Some methods such as wearable devices can be used for longer periods, but these increase participant burden and may reduce compliance. Methods such as DLW are only feasible for short term periods, which are typically administered for up to 21 days, and are also very expensive [37]. Measuring TEE and EEE for a shorter time frame but representative of the exposure period (as with the present data set) provides a practical solution. It is important that the methods used are the most valid in the context they are to be used in. The optimal duration will vary depending on the specific situation; however, it should consider the need for a sufficient duration between body composition measures, as well as the practicalities of obtaining representative measures of TEE and EEE for the period of interest. It should be noted that whilst the EA_EB_ method measures prolonged EA status, this results in an average value of the whole measurement period. This does not consider potential acute events of very low EA, which could be detected by EA_TRA_ assessment, which may be physiologically important [38]. Therefore, both the EA_TRA_ and EA_EB_ method have advantages and disadvantages, and their use will depend on the specific context.

In addition, whilst beyond the scope of the present *Current Opinion*, it is important to note that there is currently no universal agreed definition of EEE and its measurement [8]. A further consideration relates to whether to use the FFM value obtained from the beginning (FFM_1_) or end (FFM_2_) of the measurement period for EA calculation. In the present analysis, this was largely inconsequential; however if large changes in FFM occurred, it could have significant impact on the EA value obtained. Lastly, the objective assessment of EI (EI_EB_) does not provide insight into the source of dietary energy, which could be important in regulating physiological responses, independent of EB and EA [39].

## Conclusion

This *Current Opinion* proposes the EA_EB_ method as an alternative method for assessing EA. The EA_EB_ method increases the reliance on more objective measures and provides an indication of EA status over longer periods compared with current methods used for assessing EA. Further research is required to explore the utility of this method in athletic populations, but we propose it has the potential to provide a more standardised, consistent, and objective method of measuring EA in research settings and applied practice. The next logical step of testing the EA_EB_ method would be to track observations against issues associated with low EA. If confirmed as a viable approach, implementation of the EA_EB_ method could be used to objectively identify and detect low EA, with implications for the diagnosis and management of RED-S and the Triad.

## Data Availability

Available from the corresponding author upon request.

## Abbreviations

EA_EB_ method: Energy Availability - Energy Balance method
RED-S: Relative energy deficiency in sport
EA: Energy availability
EI: Energy intake
EB: Energy balance
EEE: Exercise energy expenditure
FM: Fat mass
FFM: Fat free mass
LEAF-Q: Low energy availability in females questionnaire
DXA: Dual Energy X-ray Absorptiometry
DLW: Doubly labelled water
∆ES: Change in body energy stores
TEE: Total energy expenditure
MVPA: Moderate-to-vigorous physical activity
EI_TRA_: Energy intake traditional method
EI_EB_: EI calculated using EB method
EA_TRA_: Energy availability traditional method
